# Molecular Diversity of Sapovirus Infection in Outpatients Living in Nanjing, China (2011–2013)

**DOI:** 10.1155/2016/4210462

**Published:** 2016-08-31

**Authors:** Hong-ying Zhang, Meng-kai Qiao, Xuan Wang, Min He, Li-min Shi, Guo-xiang Xie, Hei-ying Jin

**Affiliations:** ^1^Nanjing Municipal Center for Disease Control and Prevention, Nanjing 210003, China; ^2^Department of General Surgery, International Hospital of Pekin University, Beijing 102206, China

## Abstract

*Aim*. To gain insight into the molecular diversity of sapovirus in outpatients with acute gastroenteritis in Nanjing, China.* Methods*. The specimens from outpatients clinically diagnosed as acute gastroenteritis were detected by real-time PCR; RT-PCR was then performed to amplify part of VP1 sequences. The PCR products were cloned into pGEM-T Easy vector and bidirectionally sequenced. All sequences were edited and analyzed. A phylogenetic tree was drawn with the MEGA 5.0 software.* Results*. Between 2011 and 2013, 16 sapovirus positive cases were confirmed by real-time PCR. The infected cases increased from two in 2011 and six in 2012 to eight in 2013. The majority was children and the elderly (15, 93.75%) and single infections (15, 93.75%). Of the 16 real-time positive specimens, 14 specimens had PCR products and the analysis data of the 14 nucleic sequences showed that there was one GI genogroup with four genotypes, two GI.2 in 2011, three GI.2, and one GI.1 in 2012 and one GI.2, three GI.1, two GI.3, and two GI.5 in 2013.* Conclusion*. Our data confirmed continuous existing of GI genogroup and GI.2 genotype from 2011 to 2013 in Nanjing and the successive appearance of different genotypes from outpatients with gastroenteritis.

## 1. Introduction

Human calicivirus (HuCV) consists of noroviruses (NoVs) and sapovirus (SaV); they are important pathogens that are involved in nonbacterial acute gastroenteritis [1–3]. In a previous study our lab showed that HuCV has become the main pathogen of nonbacterial acute gastroenteritis in Nanjing after 2012 and replaced rotavirus as the predominant pathogen in 2013 [4].

HuCV, members of the family Caliciviridae, have 7.5-kb to 7.7-kb single-stranded genome of positive-sense RNA, which contains two or three open reading frames (ORFs). NoVs have three ORFs; ORF2 codes for the major capsid protein (VP1) with the highest degree of sequence variability in the genome. SaV has two ORFs and ORF1 codes for VP1, which also has a high degree of sequence variability. VP1 is the most important protein with diversity. We have analyzed the strain diversity of 75 strains of NoVs, genogroup II from 2010 to 2013 in Nanjing, and have found evolutionary evidence for the emergence of new GII.4 subclusters (2012 Sydney/AU) that gradually displaced previous GII.4 viruses in the population (2006b). Other scholars have also studied the strain diversity of sapovirus and found continuous existence of a single genotype in one region and successive appearance of genomically diverse sapovirus strains from patients with gastroenteritis in other countries or regions [5, 6]. However, very little is known about the circulating genotype or genomical diversity of sapovirus in Nanjing, China.

To understand the genomical diversity of sapovirus among sporadic cases in Nanjing, we analyzed sporadic sapovirus specimens collected by Nanjing Municipal Center for Disease Control and Prevention from April 2011 to October 2013.

## 2. Materials and Methods

### 2.1. Real-Time RT-PCR

Viral RNA was extracted as described in a previous paper [4]. For detecting human sapovirus, the primers and probe targeted a conserved region of the RNA polymerase [7], which was used in 25 *μ*L reaction volume with the Invitrogen Superscript III One-Step q-RT-PCR System in ABI 7500 FSAT SDS as described in a previous report [4]; the amplification results were determined, as in the same report [4].

### 2.2. RT-PCR and Sequencing of Sapovirus

A 358 to 364-nucleotide (nt) region of the 5′ end of the VP1 gene of 14 strains was amplified with primer set S1 and S2 using the Invitrogen SuperScript III One-Step RT-PCR System (Invitrogen Inc., Carlsbad, CA., USA). The forward primer S1 (5′-TA GTG TTT GAR ATG GAG GGY-3′) and reverse primer S2 (5′-CGG RCY TCA AAV STA CCB CC-3′) targeted positions 5159 to 5516 of reference strain X86560.1, Sapovirus-Manchester. The reaction was conducted with an initial RT step at 50°C for 30 mins, followed by PCR activation at 94°C for 2 mins and then 35 cycles of amplification (15 s at 94°C, 30 s at 48°C, and 30 s at 68°C) and a final extension step for 5 mins at 68°C in a GeneAmp PCR system 9700 thermal cycler (Applied Biosystems, Foster City, CA, USA). The RT-PCR products were purified, cloned, and sequenced as described in a previous report [4]; one direction sequencing entirely covered another direction.

### 2.3. Sequence Edit and Analysis

All sequences generated in this study were edited and analyzed as noted in a previous report [4]. A phylogenetic tree was drawn with the software MEGA 5.0 [8]. For the MEGA analysis, the neighbor-joining method [9] was used as the statistical method; 10,000 replicates were tested for bootstrap analysis [10]. The evolutionary distances were computed using the Kimura 2-parameter method [11] and are in units of the number of base substitutions per site. The translated amino acid sequences were aligned using the Clustal W method algorithm in the MEGA 5.0 (available at: http://mega.software.informer.com/5.0/). Phylogenetic trees were displayed with Tree-View software (available at http://softadvice.informer.com/Treeview_32_Free_Download.html).

### 2.4. GenBank BLAST Search for Additional SaV GI, GII, GIV, and GV Sequences

To compare GI.1 sequences, GI.2 sequences, and GI.3 sequences from our study with reference sequences that have been detected globally, a GenBank BLAST search was conducted with parts of VP1 sequences that were generated in this study (BLAST; http://www.ncbi.nlm.nih.gov/BLAST/) [12, 13]. Reference sequences from the NCBI website (US National Library of Medicine National Institutes of Health; http://www.ncbi.nlm.nih.gov/) were selected for drawing the phylogenetic tree; they are Sapovirus-Manchester (X86560), Sapovirus Parkville (U73124), and others as shown in the phylogenetic tree.

### 2.5. Nucleotide Sequence Accession Numbers

The VP1 sequences identified in this study were submitted to GenBank and have been assigned accession numbers, KM282587–KM282600.

## 3. Results

### 3.1. Single Genogroup and Successive Appearance of Different Genotypes of SaVs among Outpatients in Nanjing 2011–2013

Between 2011 and 2013, the Nanjing Municipal Center for Disease Control and Prevention confirmed 2, 6, and 8 sapovirus positive cases by real-time RT-PCR, respectively. For these positive cases, the majority (15, 93.75%) was single infections and only 1 case (6.25%) was coinfected with rotavirus. There were 15 cases (93.75%) of children and the elderly, including 13 children younger than 5 years old and 2 adults older than 60 years old; there was 1 case (6.25%) of an adult between 18 to 60 years old. Of the 16 real-time positive specimens, PCR products were obtained from 14 specimens and nucleic sequences were determined. Among the 14 strains, there was one genogroup GI with four genotypes, including four GI.1, six GI.2, two GI.3, and two GI.5. There was only one genotype, two GI.2 in 2011. There were two genotypes, three GI.2, and one GI.1 in 2012. In 2013, there were four genotypes, one GI.2, three GI.1, two GI.3, and two GI.5.

### 3.2. Phylogenetic Relationships among SaV Strains

**Figure 1:**
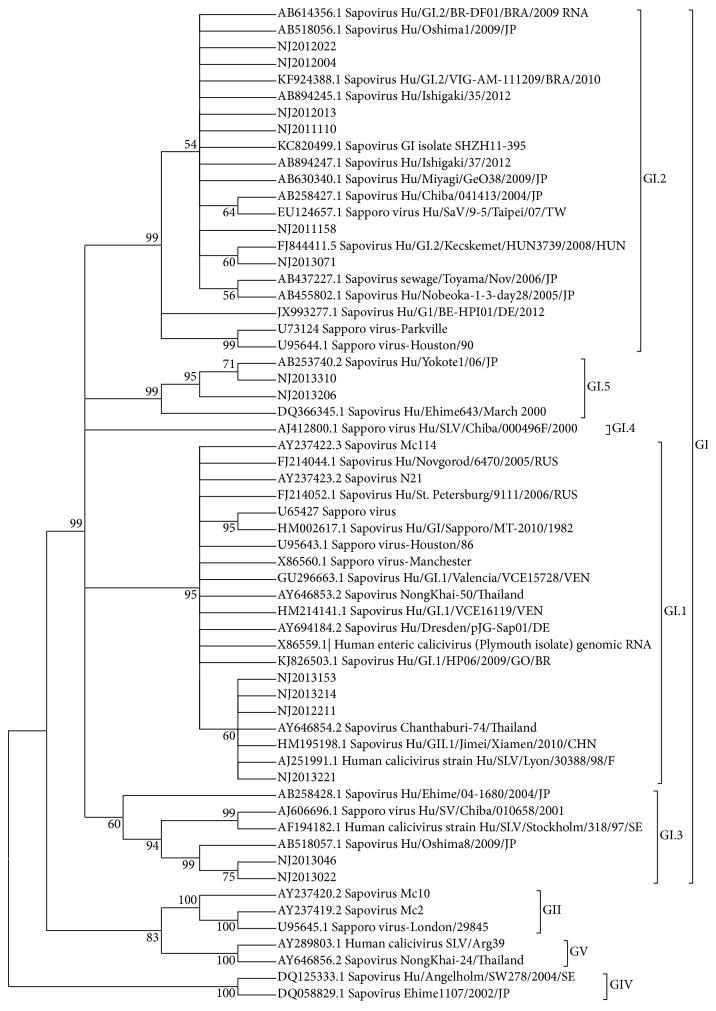
Phylogenetic analysis of the partial N terminal capsid gene (358 bp to 364 bp) of SaV strains identified in outpatients in Nanjing, China, between April 2011 and October 2013. The tree was constructed on the basis of the Kimura 2-parameter and neighbor-joining methods with MEGA5 software (http://www.megasoftware.net/) as described in Methods. The analysis involved 60 nucleotide sequences, 14 strains found in Nanjing presented as the year of detection and strain number, and 46 additional worldwide sequences presented with the NCBI accession number and details. GI, GII, GVI, and GV were genogroups, and GI.1, GI.2, GI.3, GI.4, and GI.5 were genotypes.

The phylogenetic analysis using the 358 bp to 364 bp nucleic acid sequences of 14 strains of HSVs showed different genotype diversity during different years and wide distribution of the same genogroup GI. For example, the 5 GI.2 strains from 2011 to 2013 in Nanjing had nucleotide identity level of 98%–100% between each other, 94%–100% compared to those of 15 GI.2 reference strains, and 94%–96% compared to two reference strains U95644.1∣Sapporo virus-Houston/90 and U73124∣Sapporo virus-Parkville. However, 4 GI.2 strains of 2011 and 2012 had nucleotide identity level of 99%-100% compared to outbreak strains from Japan, AB518056.1∣Sapovirus Hu/Oshima1/2009/JP, AB894245.1∣Sapovirus Hu/Ishigaki/35/2012, and AB894247.1∣Sapovirus Hu/Ishigaki/37/2012, outbreak strains from Taiwan, EU124657.1∣Sapporo virus Hu/SaV/9-5/Taipei/07/TW, and sporadic strains from Brazil, AB614356.1∣Sapovirus Hu/G1.2/BR-DF01/BRA/2009 and KF924388.1∣Sapovirus Hu/G1.2/VIG-AM-111209/BRA/2010; and 1 GI.2 strain of 2013 had nucleotide identity level of 99% compared to outbreak strains from Hungary, FJ844411.5∣Sapovirus Hu/G1.2/Kecskemet/HUN3739/2008/HUN. The 4 GI.1 strains of 2012 and 2013 had nucleotide identity level of 96%–99% compared to those of 17 GI.1 reference strains and 98-99% compared to three strains, AJ251991.1∣Human calicivirus strain Hu/SLV/Lyon/30388/98/F (98%), HM195198.1∣Sapovirus Hu/GII.1/Jimei/Xiamen/2010/CHN (99%), and AY646854.2∣Sapovirus Chanthaburi-74/Thailand (98%). Another example, the GI.3 strains of 2013 had nucleotide identity level of 90%–99% compared to 3 GI.3 reference strains, 99% compared to outbreak strain AB518057.1∣Sapovirus Hu/Oshima8/2009/JP, and 90%-91% compared to other three reference strains. For the 2 GI.5 strains of 2013, they had nucleotide identity level of 99% between each other, and 98% compared to strain AB253740.2∣Sapovirus Hu/Yokote1/06/JP, and 97% compared to strain DQ366345.1∣Sapovirus Hu/Ehime643/March 2000 (Figure 1).

### 3.3. Molecular Diversity of SaVs GI Genotypes in Nanjing

**Figure 2:**
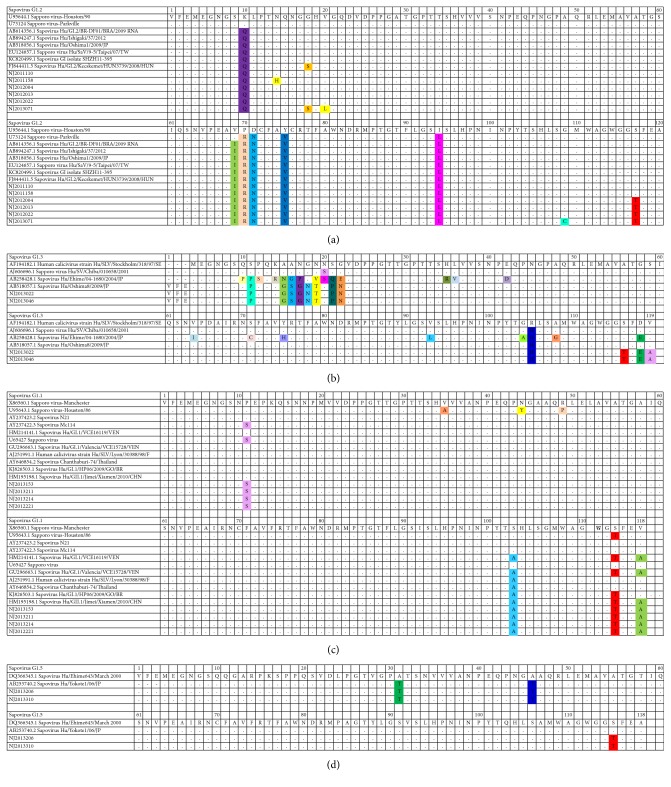
Amino acid substitutions of Nanjing strains compared to reference strains of (a) Sapovirus G1.2, (b) Sapovirus G1.3, (c) Sapovirus G1.1, or (d) Sapovirus G1.5, respectively. GI.1, GI.2, GI.3, and GI.5 were genotypes. Different colors were used for marking different substitutions in each genotype.

Sapovirus GI.2 genotype was continuous existing throughout 2011, 2012, and 2013. The substitution specific for Nanjing strains was located at S406T (Figure 2, in red), according to the location of the reference sequence of Sapporo virus-Houston/90. It seemed that there was an accumulated variation for the GI.2 genotype in 2013, with at least one more substitution compared to other reference strains (Figure 2(a)). In 2013, more genotypes merged and there were more substitutions. In addition, except for ten substitutions existing in other strains, the Nanjing GI.3 strains had two more substitutions located at S113T (Figure 2(b), in red) and V116A (Figure 2(b), right bottom, in pink), according to the location of the sequence of reference strain Hu/SLV/Stockholm/318/97/SE. The Nanjing G1.1 genotype had no specific substitution; however four substitutions P1817S (Figure 2(c), in pink), S1819A (Figure 2(c), in blue), S1831T (Figure 2(c), in red), and V1834A (Figure 2(c), in green) apparently existed in other strains, like an accumulation, according to the location of the genome for Sapovirus-Manchester. The Nanjing G1.5 genotype had a substitution located at S1831T (Figure 2(d), in red), according to the location of the reference sequence of Sapovirus Hu/Ehime643/March/2000. There seemed to be more diversity among GI.3 genotype than among other genotypes. In short, of the 14 sapovirus Nanjing strains, there was one specific substitution of S to T located at the fourth amino acid from bottom (Figure 2, in red).

### 3.4. Conserved or Variable Sites of HSVs

**Figure 3:**
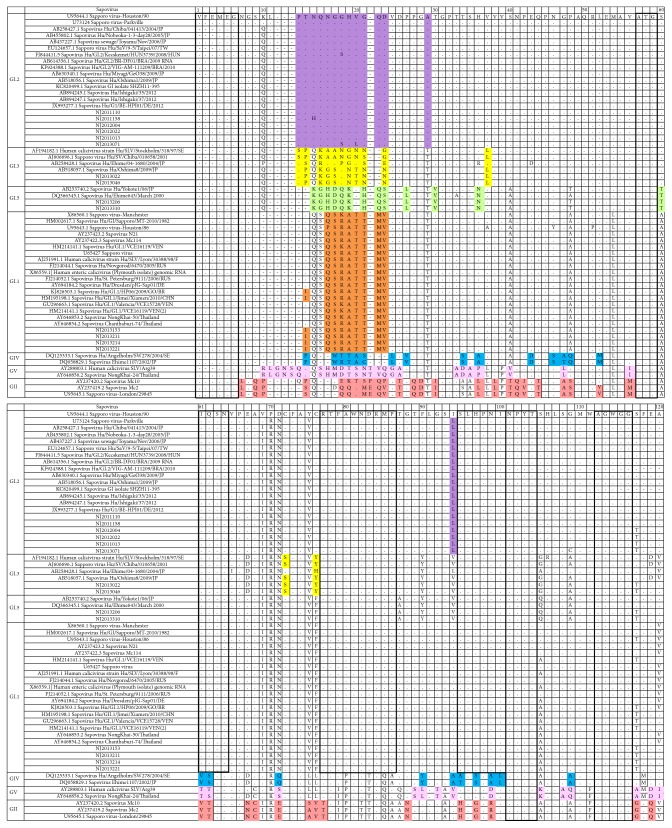
Conserved or variable sites of HSVs and Nanjing strains. The boxes were the conserved sites longer than two amino acids; some were conserved for all HSVs, including “VFEMEG,” “ATG,” “NPYT,” and “AGWGG”; others were conserved sites for genotype GI, including “IQSN,” “RTFAWNDRMP,” and “SLHPNI.” Colors were marked for the variable sites between 10 and 30 amino acids. GI, GII, GVI, and GV were genogroups, and GI.1, GI.2, GI.3, and GI.5 were genotypes.

Were there conserved or variable sites among different human sapovirus genogroups? To answer this question, we further compared the 14 sequences together with other sequences of four genogroups, GI, GII, GIV, and GV. The conserved two groups of sites were marked as boxes. First group was conserved for all HSVs, including four sites longer than two amino acids, which were “VFEMEG,” “ATG,” “NPYT,” and “AGWGG” as marked in the box. Another group was conserved in the same genogroup. For genotype GI, they were “IQSN,” “RTFAWNDRMP,” and “SLHPNI” as shown in the box. The variable sites were marked by colors. There is a most variable site between 10 and 30 for all genogroups, since they were not only different within GI genogroup, but also different among GIV, GV, or GII genogroups, as showed in Figure 3.

## 4. Discussion

Our previous studies [4] showed that rotavirus and HuCV accounted for the majority of pathogens for outpatients, about ninety percent each year from 2010 to 2013 in Nanjing; HuCV replaced rotavirus as the predominant pathogen in 2013. For cases induced by the HuCV, the majority was caused by NoVs of genogroup II; the minority was caused by SaV. Our results also showed evolutionary evidence for the emergence of new GII.4 subclusters (2012 Sydney/AU) that gradually displaced the previous GII.4 viruses in the population (2006b) [4]. In the present study, we further analyzed SaV infection and found that the majority of cases were a single infection in children and the elderly. These results were similar to other countries or regions [5–7]. Only one genogroup of GI was found in Nanjing; however, the genotype changed from the unique GI.2 in 2011 to two types GI.2 and GI.1 in 2012 to four types GI.2, GI.1, GI.3, and GI.5 in 2013. This finding implies continuous existence of GI genogroup and GI.2 genotype in Nanjing, consistent with other reports from Japan that certain genotypes can last for long time in one region and that there is successive appearance of genomically diverse SaV strains from outpatients with gastroenteritis [5, 6].

Our results are consistent with those from similar studies. Although the infection of the SaVs was much less common than that of NoVs, the SaVs were distributed broadly. The infection cases of SaVs in Nanjing outpatients were totally 16 from 2011 to 2013, including 2 from 358 cases during 2011, 6 from 310 cases during 2011, and 8 from 375 cases during 2013. The 14 strains of HSVs analyzed in this paper in Nanjing had higher nucleotide identity to strains from Japan, Brazil, Hungary, and other cities in China compared to other references strains. An investigation from 2006 to 2007 in nine provinces in China found 10 SaVs strains in six regions, one GI.1 in Guangxi, one GI.1 in Hainan, one GI.1 in Shanxi, two GI.1 and one GI.3 in Hebei, one GI.1 and one GI.3 in Jilin, and one GI.1 and one GII.3 in Shanghai [14]. Other investigations showed that there were GI.1, GI.2, and GII.1 in Hangzhou during 2009 and 2010 [15]; GI.2 and GII in Shenzhen during 2011 [16]; GI.1, GI.2, and GII.3 in Shanghai during 2011 and 2013 [17]. Further studies are needed to better understand the geographical distribution of SaVs [18].

Despite the limited studies on SaV compared to NoVs, there are some significant advances achieved by use of genotyping [19] and the epidemiologic differences between inpatients and outpatients [20]. SaV capsid sequences have been analyzed for identification of the cleavage sites or proteolytic processing [21, 22]. To understand the amino acid changes of SaV strains in Nanjing, the translated amino acid sequences of 119–121 amino acids were compared to the reference strains and different amino acid substitutions were found in the Nanjing strains. In addition, conserved and varied sites were analyzed within GI genogroup or among four genogroups, GI, GII, GIV, and GV. Whether these substitutions are involved in the antigenic changes or the virus fitness to hosts and whether the virus escaped from host recognition require further investigation. Moreover, whether the conserved or varied sites are devoted to antigenic specificity also requires further study.
